# Acute ablation of PERK results in ER dysfunctions followed by reduced insulin secretion and cell proliferation

**DOI:** 10.1186/1471-2121-10-61

**Published:** 2009-09-04

**Authors:** Daorong Feng, Jianwen Wei, Sounak Gupta, Barbara C McGrath, Douglas R Cavener

**Affiliations:** 1 Department of Biology, Pennsylvania State University, University Park, PA 16802, USA

## Abstract

**Background:**

A deficiency in *Perk *(EIF2AK3) causes multiple neonatal defects in humans known as the Wolcott Rallison syndrome. *Perk KO *mice exhibit the same array of defects including permanent neonatal diabetes (PND). PND in mice was previously shown by us to be due to a decrease in beta cell proliferation and insulin secretion. The aim of this study was to determine if acute ablation of PERK in the 832/13 beta cells recapitulates these defects and to identify the primary molecular basis for beta cell dysfunction.

**Results:**

The INS1 832/13 transformed rat beta cell line was transduced with a dominant-negative *Perk *transgene via an adenoviral vector. *AdDNPerk*-832/13 beta cells exhibited reduced expression of *insulin *and *MafA *mRNAs, reduced insulin secretion, and reduced cell proliferation. Although proinsulin content was reduced in *AdDNPerk*-832/13 beta cells, proinsulin was abnormally retained in the endoplasmic reticulum. A temporal study of the acute ablation of *Perk *revealed that the earliest defect seen was induced expression of two ER chaperone proteins, GRP78/BiP and ERp72. The oxidized states of ERp72 and ERp57 were also increased suggesting an imbalance in the redox state of the ER.

**Conclusion:**

Acute ablation of Perk in INS 832/13 beta cells exhibited all of the major defects seen in *Perk KO *mice and revealed abnormal expression and redox state of key ER chaperone proteins. Dysregulation of ER chaperone/folding enzymes ERp72 and GRP78/BiP occurred early after ablation of PERK function suggesting that changes in ER secretory functions may give rise to the other defects including reduced insulin gene expression, secretion, and cell proliferation.

## Background

Monogenic forms of permanent neonatal diabetes have revealed key aspects of the development and function of the insulin secreting beta cells [1]. Mutations in *Perk *(EIF2AK3) underlies the complex genetic disorder of the Wolcott Rallison syndrome, which includes permanent neonatal diabetes (PND), exocrine pancreas deficiency, growth retardation, hepatic dysfunctions, and skeletal dysplasias [2,3]. All of the dysfunctions in WRS are mirrored in mice deficient for PERK [4,5], and detailed genetic studies in mice have shown that the diabetes is caused by the loss of expression of PERK in the insulin secreting beta cells [6], whereas the exocrine pancreas deficiency is caused by the absence of PERK in the pancreatic acinar cells [7]. The initial interpretation of the molecular basis of these dysfunctions in humans and mice was based upon studies performed in vitro with cultured fibroblasts [8]. These studies showed that the catalytic domain of PERK resides in the cytoplasm where it phosphorylates the translation initiation factor eIF2 alpha, which results in either repression of global protein synthesis or activation of translation of specific mRNAs encoding gene regulatory proteins. PERK regulatory domain resides in the lumen of the ER and is controlled by the binding of the ER chaperone proteins, GRP78/BiP and GRP94, and calcium [9]. Disturbances in the ER such as ER stress and accumulation of unfolded proteins, or normal physiological changes in calcium levels can activate PERK [9,10]. Based upon these studies it was proposed that PND in humans and mice deficient in PERK was caused by uncontrolled ER stress and apoptotic cell death of the beta cells [11]. However a recent comprehensive analysis of the islet and beta cell development revealed that the cause of diabetes in *Perk *deficient mice is due to failure to expand beta cell mass and defects in beta cell development and insulin secretion during the critical fetal and neonatal periods [6]. Thus three distinct defects are seen in *Perk*-deficient beta cells, which raises questions about the causal connection and progression of beta cell dysfunction. The chronic loss of PERK expression over the four-week period between when defects in beta cell development are first seen during the fetal stage and the onset of overt diabetes three weeks after birth confounds resolving these questions and determining the molecular basis of the defects in *Perk*-deficient mice. To investigate the acute effects of PERK ablation we have expressed a dominant negative mutation of *Perk *in the transformed INS1 832/13 beta cell line. We found that acute ablation of PERK in 832/13 beta cells mimics all of the defects seen in the beta cells of *Perk*-deficient mice and detailed temporal studies suggest that defects in the function of the endoplasmic reticulum may give rise to the defects in beta cell developmental and proliferation.

## Results

### Ablation of PERK in INS1 832/13 beta cells results in reduced insulin gene expression, insulin content, and insulin secretion

**Figure 1:**
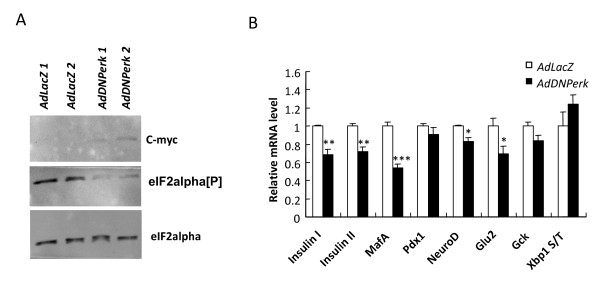
**Expression of *AdDNPerk *in 832/13 cells down-regulates insulin mRNA levels**. *A*. Expression of DNPERK was detected by tag c-myc antibody and the eIF2α [P]/total eIF2α was reduced when two different multiplicities of infection were used. (MOI *of AdLacZ1 and AdDNPerk1 *= 10; MOI *of AdLacZ2 and AdDNPerk2 *= 20). *B*. At 48 hr post-transduction, the expression of insulin gene, *MafA*, *NeuroD*, and *Glu2 *was significantly reduced in *AdDNPerk-832/13 *cells. The data are expressed relative to the *AdLacZ*-832/13 (= 100%) ± SE, n = 4. *P < 0.05, **P < 0.01, ***P < 0.001.

**Figure 2:**
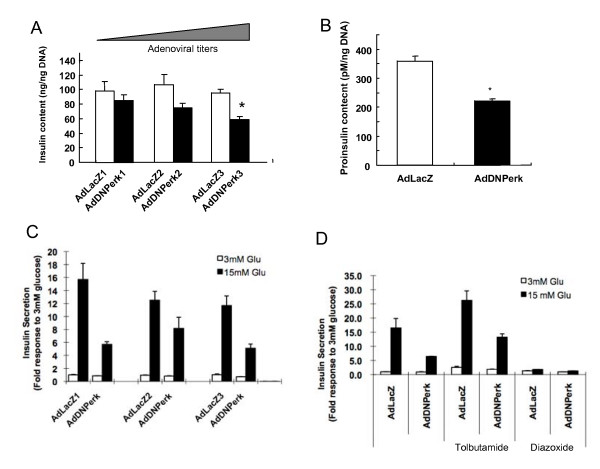
**Ablation of PERK by *AdDNPerk *in 832/13 cells results in reduced insulin and proinsulin content and impaired insulin secretion**. ***A***. At 48 hr post-transduction, insulin content was assayed by ELISA in cells infected with *AdDNPerk *and control virus at 5, 10 and 20 MOI. The data were normalized to DNA content. ***B***. Proinsulin content was assayed by ELISA and normalized to DNA content. ***C***. Insulin secretion in response to 3 and 15 mmol/l glucose was measured in cells infected with recombinant virus at 5, 10 and 20 MOI. ***D***. Insulin secretion in response to 3 and 15 mmol/l glucose with or without 200 μmol/l tolbutamide or 250 μmol/l diaxoxide. Insulin secretion in response to 15 mM glucose in C and D was normalized to total insulin content, and was expressed as a fold increases relative to insulin secreted from the control *AdLacZ-832/13 *cultured in 3 m mol/l glucose. Values are means and error bars are ± SE, n = 3, *p < 0.05.

To investigate the acute effects of ablating PERK expression, the transformed rat INS 832/13 insulin-secreting beta cells [12] were transduced with a dominant negative mutant of *Perk *using the adenoviral vector (*AdDNPerk*) yielding *AdDNPerk*-832/13 beta cells. The *AdDNPerk *transgene contains a C-terminal truncation of the kinase domain of mouse *Perk *[13] tagged with c-myc which was inserted into an adenoviral vector that contains a GFP marker to monitor transduction efficiency. An adenoviral vector expressing the *E. coli *lacZ (β-galactosidase) denoted *AdLacZ *was used throughout as a control. After 24-48 hours post-transduction of *AdDNPerk*, greater than 90% of the 832/13 beta cells were successfully transduced as shown by expression of the GFP marker (not shown). The DNPERK protein was detected (Figure 1A) in transduced 832/13 beta cells, and significantly ablated the activity of the endogenous PERK as shown by reduced levels of phosphorylated eIF2 alpha (Figure 1A). Ablation of PERK resulted in a significant reduction of the *insulin-1*, *insulin-2*, *MafA*, and *Glut2 *mRNAs while the expression of other important genes in beta cell development and function including *Pdx-1*, *NeuroD*, and *Gck *were nearly normal (Figure 1B). In parallel to decreased *Insulin-1 *and *Insulin-2 *mRNA expression, insulin content per beta cell was significantly reduced as a function of the dose of DN-PERK transduced in the 832/13 beta cells (Figure 2A, B) mirroring the reduced insulin content of the pancreatic islets of *Perk KO *mice [6].

Islets isolated from neonatal *Perk KO *mice exhibited an almost complete ablation of glucose-stimulated insulin secretion (GSIS) [6]. To determine if an acute deficiency of PERK impacts insulin secretion, 832/13 beta cells were transduced with *AdDNPerk*. Forty-eight hours post-transduction of *AdDNPerk*-832/13 cells showed a 3-fold reduction in insulin secretion in response to 15 mM glucose stimulation compared to 832/13 transduced with the *AdLacZ *control (Figure 2C). Treatment of *AdDNPerk*-832/13 cells with tolbutamide, a KATP channel blocker, also showed reduced enhancement of glucose stimulated insulin secretion compared to the control cells. Treatment with diazoxide, a KATP channel opener, blocked glucose-stimulated insulin secretion (Figure 2D) in both genotypes as expected. We conclude that the reduced GSIS is due to a combination of decreased insulin content as well from a partial reduction in KATP-dependent insulin secretion.

### Proliferation of *AdDNPerk *832/13 beta cells is reduced

**Figure 3:**
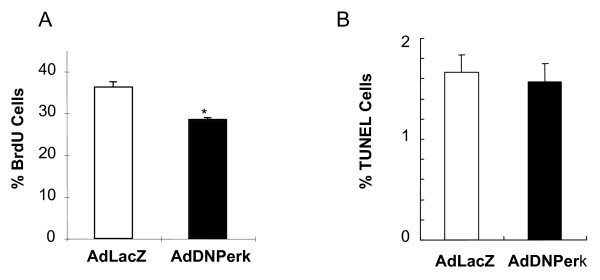
**Ablation of PERK in 832/13 cells impairs beta cell proliferation but does not increase cell death**. *A. BrdU *incorporation is significantly reduced in cells infected with *AdDNPerk *at 24 hr post-transduction cells compared to *AdLacZ *infected cells. The data represent two thousand cells for each treatment in two independent experiments (*p < 0.05). *B*. Cell Death. The percentage TUNEL-positive 832/13 cells infected with the *AdDNPerk *was low and was not significantly different than the cells infected with the *AdLacZ *control. A MOI of 20 was used for these experiments.

The cell proliferation rate of *AdDNPerk*-832/13 and LacZ-832/13 beta cells was estimated by BrdU incorporation. *AdDNPerk*-832/13 exhibited a 25% reduction in proliferation compared to *AdLacZ*-832/13, consistent with a general observation of the relative growth rates of these cells across several independent experiments (Figure 3A). Cell death, estimated as TUNEL positive cells, was low in both genotypes and not significantly different from each other (Figure 3B).

### Accumulation of proinsulin in the ER occurs in the absence of increased protein synthesis in *Perk *deficient beta cells

**Figure 4:**
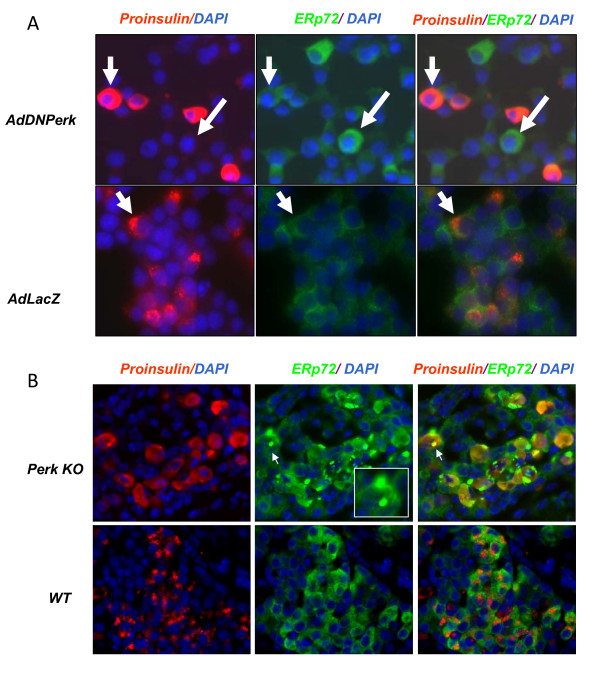
**Ablation of PERK resulted in abnormal accumulation of proinsulin and ERp72 in beta cells**. A. At 48 hr post-transduction, some of *AdDNPerk*-infected 832/13 cells had abnormal accumulation of proinsulin in the ER (upper panel, left arrow). ERp72 is upregulated in cells infected with *AdDNPerk *and the increase in ERp72 staining appeared earlier than the Impacted-ER phenotype (upper panel, right arrow). In contrast, cells infected with the *AdLacZ*-832/13 control exhibited normal Golgi localization of proinsulin (lower panel, arrow). B. ERp72 is abnormally retained in *Perk KO *beta cells in neonatal P1 mice. Double staining for both proinsulin and ERp72 demonstrates higher expression of ERp72 in beta cells than in exocrine cells and other endocrine cells in both WT and KO. In KO cells the ERp72 antibody stains balloon-like structures (shown in the inset). The data represent three or more independent mice for each genotype.

Proinsulin is co-translationally imported into the endoplasmic reticulum and rapidly enters the Golgi complex [14,15] where it accumulates in beta cells prior to its maturation and formation of insulin secretory granules [6]. In *Perk KO *mice, however, a large fraction (ca. 20-40%) of the beta cells exhibited grossly distended endoplasmic reticulum with a very high accumulation of proinsulin [6]; these abnormal cells are denoted herein as Impacted-ER beta cells. Acute ablation of PERK in 832/13 cells also resulted in appearance of Impacted-ER cells as characterized by a shift of the majority of proinsulin from the Golgi complex to the endoplasmic reticulum (Figure 4A). In contrast we have not observed Impacted-ER cells in the control *AdLacZ*-832/13 cells (Figure 4A) or in the pancreatic islets of wild-type mice (Figure 4B).

**Figure 5:**
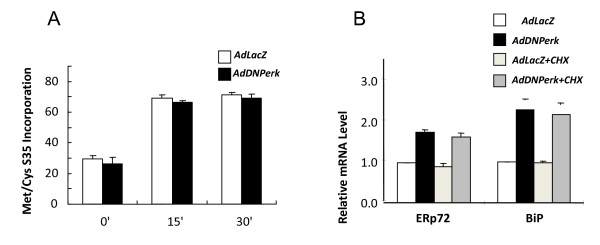
**Global protein synthesis is normal following acute ablation of PERK**. A. Incorporation of 35S translabel into total TCA-precipitable protein did not differ between *AdDNPerk*-infected and *AdLacZ*-infected cells at 24 hr (A) or 16 hr post-transduction (data not shown). Values are means ± SE from three replicate samples. B. Cyclohexamide (CHX) did not inhibit the induction of ERp72 and BiP mRNA in *AdDNPerk*-infected 832/13 cells. At 12 hr post-transduction, cells were treated with 5 mg/ml CHX for 4 hours before RNA was extracted for Real-time quantitative RT-PCR. Values are means ± SE, from three replicate samples.

When PERK is highly activated by ER stress it represses global protein synthesis [16,17], and it has been speculated that the loss of PERK function may result in derepressed protein synthesis and over accumulation of proteins in the ER. Conversely, we found that global protein synthesis rates were not elevated in *AdDNPerk*-832/13 cells (Figure 5A) and proinsulin content was reduced, not increased (Figure 2B). Thus the over accumulation of proinsulin in the ER is not due to over synthesis of proinsulin.

### ERp72 and GRP78/BiP expression are induced first after initiating acute ablation of *Perk *followed by other changes in gene expression

**Figure 6:**
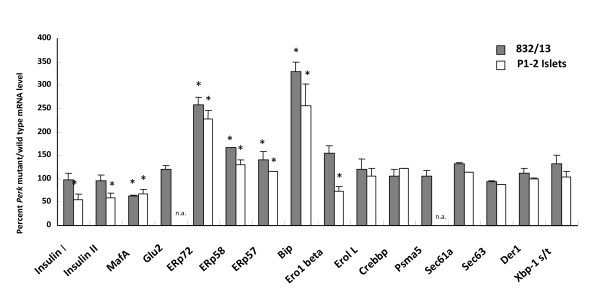
**Comparison of gene expression in Perk deficient islets and 832/13 transformed beta cells**. The expression of mRNAs in *Perk*^-/- ^islets and *AdDNPerk 832/13 *beta cells were normalized to *Perk*^+/+ ^islets and *AdLacZ 832/13 *beta cells, respectively. For the 832/13 cells a low dose of adenovirus (MOI = 5) was used, and the data from 2 to 5 independent experiments were analyzed. For islets, mRNA expression was also quantified from 5-9 WT and 4-9 *Perk KO *mice. For *Xbp-1 *the spliced *Xpb-1 *mRNA form was normalized to total *Xbp-1 *mRNA. Not assayed for specific mRNAs denoted as n.a.

To further explore the possible causes and consequences of proinsulin retention in ER in *Perk*-deficient beta cells, the expression of key ER chaperone, trafficking, ERAD genes were examined in 832/13 cells that had been transduced with a relatively low titer (10 MOI) of *AdDNPerk *and in pancreatic islets isolated from neonatal (P1) *Perk KO *mice. Using a lower titer of *AdDNPerk*, *Ins1*, *Ins2*, and *Glut2 *were expressed at normal levels (Figure 6), unlike that seen at high titers (Figure 1B) and *Perk KO *islets where these genes were significantly repressed compared to control. *MafA *mRNA was significantly repressed in both *AdDNPerk*-832/13 and *Perk KO *islets. The ER chaperone genes *ERp72 *and *GRP78/BiP *were substantially induced while most other ER chaperones (ERp58, ERp57, Ero1β, and Ero1L) and ERAD associated (Psma5, Sec61a, Sec63, and Der1) genes were not significantly elevated (Figure 6). To determine the temporal pattern of the induction of these genes following acute ablation of *Perk*, the mRNA level of several genes was assessed at various time points after transduction of 832/13 beta cells with *AdDNPerk *(Table 1). *Ins1*, *Ins2*, and *MafA *were not significantly reduced until 36 hours after transduction, whereas *ERp72 *and *GRP78/BiP *were induced after only 16 hours. The beta cells of *Perk KO *mice showed reduced expression of several cell-cycle and proliferation genes associated with a substantial reduction in beta proliferation [6]. *Cyclin A *and *cyclin D *mRNAs were also reduced in *AdDNPerk*-832/13 beta cells but not until 48 hours. The splicing of *Xbp-1 *mRNA is a sensitive indicator of the ER stress response, and the over accumulation of proinsulin is predicted to elicit and an ER stress response, but surprisingly neither beta cells in *Perk *deficient mice or *AdDNPerk*-832/13 beta cells showed increased splicing of *Xbp-1 *mRNA (Figure 6). Increased expression of ERp72 in *AdDNPerk-832/13 *cells was also seen by immunohistochemistry earlier than the appearance of the Impacted-ER cells (Figure 4A). We further tested the hypothesis that unrepressed global protein synthesis is responsible for the induction of the ER chaperones *ERp72 *and *GRP78/BiP *by treating *AdDNPerk*-832/13 beta cells with cyclohexamide, a potent inhibitor of protein synthesis. Cyclohexamide did not block the induction of *ERp72 *and *GRP78/BiP *in *AdDNPerk*-832/13 beta cells (Figure 5B) and did not reduce or delay the appearance of Impacted-ER beta cells (data not shown) thus arguing against this hypothesis.

**Table 1:** Temporal Changes in gene expression following Perk ablation in 832/13 cells

	**6 hr**	**16 hr**	**36 hr**	**48 hr**
*Ins1&II*	1.02 (0.00)	1.04 (0.07)	0.64 (0.05)*	0.63 (0.12)*
*MafA1*	0.91 (0.10)	0.81 (0.10)	0.49 (0.12)*	0.64 (0.14)*
*ERp72*	0.92 (0.12)	1.81 (0.16)*	2.66 (0.52)*	3.24 (1.10)*
*BiP*	1.08 (0.26)	2.04 (0.32)*	4.26 (1.25)*	2.29 (0.34)*
*Xbp1-S/T*	1.03 (0.04)	1.35 (0.22)	1.56 (0.11)	1.32 (0.19)
*Cyclin A*	1.03 (0.05)	0.91 (0.06)	0.93 (0.05)	0.74 (0.10)*
*Cyclin D*	1.22 (0.04)	1.05 (0.08)	1.07 (0.23)	0.85 (0.11)

The values represent the levels of specific mRNAs in *AdDNPerk*-transduced cells normalized to *AdLacZ*-transduced controls as determined by real-time quantitative RT-PCR. Time points (hr) indicate hours after transduction. The data are the average of two to five independent experiments. * P < 0.05

**Figure 7:**
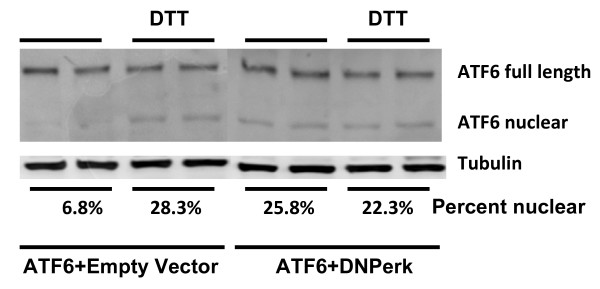
**Basal levels of processed ATF6 are elevated in PERK-ablated *cells***. *AD293 cells *were co-transfected with *EYFP-ATF6a *and either empty vector or *DNPerk*. Each type of co-transfection was treated with and without 5 mM DTT for 1 hr. Immunoblotting with an antibody that recognizes tagged ATF6 shows both the ER-resident uncleaved and cleaved nuclear forms. Quantification of the nuclear ATF6 expressed as a percentage of the full-length form is shown below the tubulin panel.

ERp72 and GRP78/BiP are induced by the transcription factor ATF6 [18]. Although we could not readily detect the active nuclear form of ATF6 in 832/13 beta cells (not shown), we examined the processing of ATF6 in the human embryonic kidney AD293 cell line co-transfected with plasmids bearing *ATF6 *and *DNPerk *or vector control and treated with DTT, an ER stress inducer. As expected, ATF6 processing was induced by DTT in control cells (Figure 7). However, in AD293 cells transfected with *DNPerk*, the processed nuclear form of ATF6 was present at relatively high levels in untreated cells and not further induced by DTT. As new protein synthesis is not required for the induction of *ERp72 *and *BiP *mRNA expression following acute ablation of *Perk*, post-translational processing of ATF6 to its active nuclear form is consistent with the hypothesis that ATF6 is responsible for their induction.

### ERp72 protein expression and oxidized state is increased in PERK-ablated 832/13 cells

**Figure 8:**
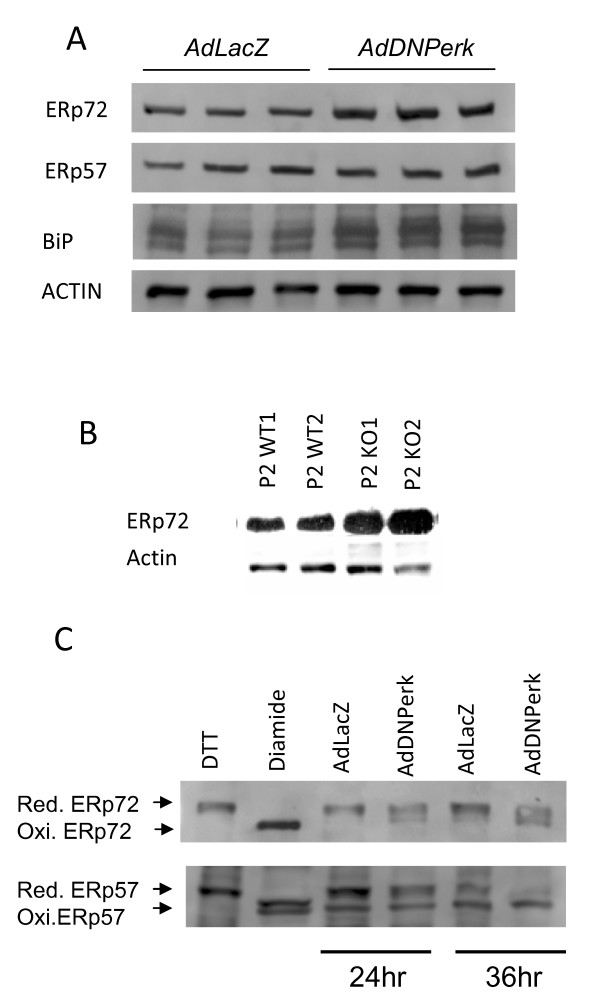
**ERp72 and GRP78/BiP protein are elevated and ERp72 oxidized state is increased in *AdDNPerk 832/13 *beta cells**. A. Triplicate samples were probed with antibodies to ERp72, ERp57, BiP and actin, which served as the loading control. B. ERp72 expression was increased in islets from P2 *Perk *KO mice. Samples are from duplicate mice for each genotype. C. At 24 hr or 36 hr post-transduction, protein samples were isolated and treated with AMS to differentiate the reduced and oxidized forms of ERp72 and ERp57 on PAGE gels. Western blots showed increased oxidized isoforms of both ERp72 and ERp57 in 832/13 cells infected with *AdDNPerk *compared to cells infected with *AdLacZ*.

ERp72, a family member of the protein disulfide isomerases (PDI), is localized in the ER and plays a major role in quality control and folding [19-22]. These PDI enzymes are particularly important for ER client proteins such as proinsulin that depend upon cysteine disulfide bonds for their folded structure. Indeed we have found that ERp72 was physically associated with proinsulin in 832/13 cells (data not shown). As seen for *ERp72 *mRNA levels, ERp72 protein was elevated in *AdDNPerk*-832/13 beta cells and similar increases were seen for GRP78/BiP (Figure 8A). In *Perk KO *mice we found that the amount of ERp72 was elevated (Figure 8B) and became localized to large balloon-like structures in Impacted-ER cells (Figure 4B). Comparison of the ERp72 localization seen in IHC images (Figure 4B) to TEM images of Impacted-ER beta cells [6] suggest that these structures correspond to one of two types of distended ER most similar to Russell bodies [23,24]. These ERp72 enriched structures were rarely observed in the *AdDNPerk*-832/13 cells.

The redox state of the PDI family of ER folding enzymes including ERp72 and ERp57 is known to vary according to changes in the oxidative and functional states of the ER. In addition to increased levels of ERp72 protein, *AdDNPerk*-832/13 beta cells exhibited an increase in the oxidized isoforms of ERp72 and ERp57 (Figure 8C) that were first seen at 24 hours post transduction and increased significantly by 36 hours.

## Discussion

Despite the vast differences between the milieu and ontogeny of cultured beta cells and native beta cells in the endocrine pancreas, we found that the array of defects in the beta cells of *Perk KO *mice [6] were remarkably present following acute ablation of *Perk *in the transformed rat INS1 832/13 beta cells. These defects include reduced cell proliferation (without changes in cell death), reduced expression of proinsulin and insulin, reduced glucose-stimulated insulin-secretion, and abnormal retention of proinsulin in the ER leading some cells to have a highly distended ER. That these defects underlie the cause of neonatal diabetes in *Perk KO *mice, and by inference in humans with the Wolcott Rallison syndrome, was shown by the study of tissue-specific *Perk KO *mice and the ability of a beta cell specific-Perk transgene to rescue diabetes [6,7,25]. Given the close correspondence of cellular pathology of the *AdDNPerk*-832/13 beta cells to the beta cells of *Perk KO *mice, we were encouraged to take advantage of cultured beta cells to elucidate the molecular function of PERK in regulating beta cell development and physiology.

Acute ablation of Perk, afforded by transduction of the dominant negative *Perk *transgene, allowed us to determine the progress and order of the ensuing defects over time. As we had seen in *Perk KO *mice, the expression of insulin synthesis genes *Ins-1*, *Ins-2*, and *MafA*) and cell cycle genes was reduced but the reduction in their expression lagged behind the induction of the ER chaperone genes *ERp72 *and *GRP78/BiP *thus suggesting that a perturbation in the ER may result in repression of genes involved in beta cell function and proliferation. Initial reports showing that loss of PERK function resulted in neonatal diabetes in humans and mice [2,4,5] suggested that the molecular defect was related to the function of PERK in the regulation of the ER stress - Unfolded Protein Response (UPR). Our extensive analyses of *Perk*-deficient beta cells in mice [6] and in *AdDNPerk*-832/13 beta cells in culture have not supported this hypothesis. The original hypothesis assumed that global protein synthesis would be derepressed as a direct consequence of the absence of PERK, and that this would result in uncontrolled protein synthesis and overloading of the ER culminating in apoptotic cell death [4]. Although the ER in *Perk*-deficient beta cells show marked accumulation of proinsulin, we found that global protein synthesis was not elevated nor was there an elevation in the proinsulin content in beta cells. Cell death did not increase, but instead cell proliferation was ablated. Moreover, the expression of *ERp72 *and *GRP78/BiP *was still induced in *AdDNPerk*-832/13 beta cells after blocking protein synthesis with cyclohexamide suggesting that their induction was not due to protein overload.

ERp72 and GRP78/BiP are positively regulated and insulin gene expression is negatively regulated by ATF6 [18,26], and we found that the processed nuclear form of ATF6 was substantially increased in PERK-ablated AD293 cells. Treatment of cells with Brefeldin-A also induces the processing of the ATF6 [27], which is caused by relocalization of the S2P protease from the Golgi to the ER. We found that the S2P protease is indeed mislocalized in PERK-ablated AD293 cells and is associated with ER markers (Gupta, et al. unpublished data), which suggests that the increased basal level of the nuclear form of ATF6 is due to mislocalization of the proteases that cleave precursor ATF6 rather than by ER stress. Further studies are required to confirm the importance of these findings in insulin-secreting beta cells.

In addition to its role in repressing global protein synthesis during ER stress, PERK positively regulates the translation of ATF4, which in turn upregulates GADD153/CHOP [8,28,29]. Mutations in these downstream genes, however, do not lead to diabetes in mice, [6,7] thus arguing against the hypothesis that diabetes associated with *Perk *deficiency is related to the role of PERK in regulating the unfolded protein response. Considerable evidence suggests that PERK is important for mediating the ER stress response in adult beta cells [11,30-32], but we argue that the primary function of PERK in beta cells is to regulate beta cell development and function during the fetal-neonatal period. Interestingly IRE1, another mediator of the ER stress response, has also been shown to have an important beta cell function by positively regulating insulin biosynthesis [33].

We speculate that the Impacted-ER phenotype, characterized by gross distension of the endoplasmic reticulum and abnormal retention of proinsulin observed in *Perk*-deficient beta cells, represents the extreme manifestation of ER dysfunction. The induction of ERp72 and GRP/78 mRNA expression is the first hint of ER dysfunction, and is followed by increased levels of these proteins and the oxidized isoform of ERp72 and ERp57. The increased oxidized isoforms of ERp72 and ERp57 suggests that redox state of the ER is abnormal or that the oxidation and reduction of protein disulfides is imbalanced. The formation of disulfide bonds is essential for the proper folding proinsulin and subsequent maturation of insulin [14]. The protein disulfide isomerase family including ERp72, PDI, and ERp57, mediate their oxidative protein folding functions by forming transient mixed disulfides with ER client proteins [34,35] such as proinsulin. In addition to catalyzing the proper folding of proteins in preparation for transport from the ER to the Golgi, the PDI enzymes participate in ER associated protein degradation (ERAD) by reducing thiols of misfolded proteins in preparation for retrotranslocation to the cytoplasm for proteosomal degradation [20,34]. Recently Forster and colleagues [19] have shown that ERp72 and PDI have opposing functions in the ER. ERp72 assists proper protein folding whereas PDI facilitates unfolding of misfolded proteins in preparation for proteosomal degradation. We propose that PERK regulates the expression of key genes that encode ER redox and protein folding functions in the insulin-secreting beta cells during the dynamic metabolic changes that occur during the developmental transitions through embryonic, fetal, neonatal, and juvenile stages. PERK enzyme activity is regulated through its ER luminal domain via binding of GRP78/BiP complexed with calcium [36,37]. Physiological changes in ER calcium modulate PERK activity [10] as well as insulin secretion. The regulation of ER calcium is coupled to the redox state of specific ER proteins including SERCA, the major ER calcium pump [38]. We speculate that PERK acts as an ER calcium sensor that couples ER functions of folding, quality control, and protein trafficking that are intimately tied to the redox state of the ER as a function of the dynamic changes in the physiological environment of the beta cell during the early developmental transitions.

## Conclusion

We found that acute ablation of PERK in the transformed 832/13 beta cell line leads to reduced proliferation and reduced insulin gene expression, insulin content, and insulin secretion, demonstrating that acute ablation of PERK recapitulates the major cellular and molecular defects seen in *Perk KO *mice. In addition proinsulin accumulates abnormally in the ER, which occurs after the elevation of two key chaperone proteins GRP78/BiP and ERp72. ERp72 accumulates in a sub compartment of the ER and its oxidized isoform is substantially increased. Because the changes in the expression of GRP78/BiP and ERp72 are the earliest changes observed after ablation of *Perk*, we suggest that ER dysfunctions give rise to defects in proinsulin trafficking, insulin secretion, and cell proliferation.

## Methods

### Cell culture

INS-1 832/13 insulin-secreting beta cells were obtained from Dr. Christopher Newgard (Duke University). The 832/13 cells were cultured in RPMI-1640 (Mediatech Cellgro) supplemented with 11 mM glucose, 10% fetal bovine serum, 10 mM HEPES, 1 mM sodium pyruvate, 50 μM β-mercaptoethanol and Antibiotic Antimycotic Solution (Sigma) at 37°C in 5% CO_2_, 95% air. The cells were sub-cultured twice weekly. For determining relative proliferation, BrdU (1 mM) was added to the cell for 1 hr followed by permeabilization and fixation for 10 minutes with 4% formaldehyde, 0.1% Triton x-100 in PBS. The cells were then denatured with 1N HCL for 30 mins, and anti-BrdU (1:50, DAKO) applied for 1 hour. For estimating cell death the DeadEndTM Fluorometric TUNEL System (Promega, Inc.) was used. Cells were fixed in 4% methanol-free formaldehyde solution for 10 min, permeabilized with 0.2% Triton X-100 solution for 5 minutes, and labeled according to instructions of the kit.

### Proinsulin content, insulin content and insulin secretion

After 48 hr post-transduction, 832/13 cell pellets were sonicated in 1 mol/l acetic acid containing 0.1% bovine serum albumin. Aliquots of cell extracts were assayed for proinsulin and insulin content using proinsulin EIA (ALPCO) and Mouse Insulin Ultrasensitive EIA (ALPCO), respectively. The data were normalized to DNA content. Insulin secretion was assayed at 3 mmol and 15 mmol/l glucose. For KATP channel dependent secretion assay, 200 μmol/l tolbutamide or 250 μmol/l diazoxide were added.

### Plasmids and adenovirus production

The c-myc tagged dominant-negative mouse *Perk *transgene (*PERKΔC*) was kindly provided by Dr. David Ron (New York University). *PERKΔC *was generated by deleting the kinase domain of Perk residing in the carboxy terminal coding sequence, retaining the amino terminal ER luminal domain (Harding, 1999). The *PERKΔC *transgene was excised by us from the parent vector by digestion with *Spe*1 and *Xho*1 and then inserted to the Adenovirus vector pShuttle-IERS-hrGFP-1 (Stratagene). The resulting construct, which we denote as *AdDNPerk*, was transfected into AD-293 cells using the MBS Mammalian Transfection kit (Stratagene) for amplification. The adenovirus was then purified by Adeno-X™ Virus Purification kit (Clontech) and the titer was determined by the QuickTiter™ Adenovirus Titer Immunoasssay Kit (Cell Biolabs, Inc.). The pShuttle-CMV-lacZ transgene, denoted herein as *AdLacZ*, served as a control. Transduction of 832/13 cells was carried out at a cell confluency of about 70%, at different multiplicities of infection (MOI) from 5 to 20. A MOI of 20 was used in most experiments unless otherwise stated. After 2 hours of incubation with the virus at 37°C, 5% CO2, cells were washed once in RPMI and cultured for an additional 6-48 h before assay.

To assess ATF6 processing the expression plasmid pCMVshort-EYFP-ATF6-alpha, provided by Kazutoshi Mori, was transfected into AD293 cells.

### Mouse strains

The *Perk *knockout strain was previously generated by us [5] and are congenic for C57BL/6J or 129SvEvTac. Both congenic strains exhibited all of the beta cell defects previously described [5].

### Islet isolation

The pancreata from E18.5 to p2 mice were inflated in situ by injecting 3 mg/ml Collagenase P (Invitrogen) in HBSS (Sigma) and digestion was carried out at 37°C for about 12 mins at which time 1 ml of ice-cold HBSS was added to stop the enzyme reactions. The dispersed pancreatic tissue was pelleted and washed once with HBSS. The pellets were then suspended in 1 ml Histopaque-1077, overlaid with 0.2 ml RPMI 1640 medium and centrifuged at 890 g for 12 min to separate the islet from the other cell types and residual debris. Islets were manually picked under a dissection microscope and washed once with ice-cold HBSS.

### Gene expression levels

Quantification of gene expression was carried out by using qPCR Core Kit for SYBR Green I (Eurogentec) amplifying cDNA with the ABI Prism 7000 Sequence Detection System. The following cycling conditions were used for all primer pairs: 50°C (2 minutes), 95°C (10 minutes), 40 cycles of (95°C [15 seconds], 60°C [1 minute]). Levels of *Xbp1-s *(spliced form) were normalized to *Xbp1-t *(total) levels. All other mRNAs were normalized to the levels of actin and/or GAPDH. The mouse primer sequences were as follows: *actin 5'-GCCCTGAGGCTCTTTTCC-3', 5'-TGCCACAGGATTCCATACCC-3'; GAPDH 5'-GGAGCGAGACCCCACTAACA-3', 5'-ACATACTCAGCACCGGCCTC-3'; Insulin I 5'-AGCATCTTTGTGGTCCCCAC-3', 5'-CCCCACACACCAGGTAA-3'; Insulin II 5'-CAGAAGCGTGGCATTGTAGA-3', 5'-TTGCAGTAGTTCTCCAGCTGG-3'; MafA, 5'-GCTGGTATCCATGTCCGTGC-3', 5'-GTCGGATGACCTCCTCCTTG-3'; Pdx-1, 5'-GAGCGTTCCAATACGGACCA-3',5'-TCAGCCGTTCTGTTTCTGGG-3'; ERP72, 5'-TTCCACGTGATGGATGTTCAG-3', 5'-AGTCTTACGATGGCCCACCA-3'; ERP57, 5'-GGCGGATGCAACATATCACC-3', 5'-TGTGGTTCGTACTGTCCCCC-3'; ERP58, 5'-CAAGAGGCTTGCCCCTGAG-3', 5'-GGTGTTTGTGTTGGCAGTGC-3'; Hrd1, 5'-TGGCTTTGAGTACGCCATTCT-3', 5'-CCACGGAGTGCAGCACATAC-3'; Ero1 L, 5'-AAACCCTGCCATTCTGATGAA-3', 5'-ACTCATCCACGGCTCCAA GT-3'; Ero1 beta, 5'-TGATTCGCAGGACCACTTTTG-3', 5'-TAGCCAGTGTACCGTTCCGG-3'; Herpud1, 5'-CCCACCTGAGCCGAGTCTAC-3', 5'-CTTGGAGACACTGGTGATCCAA-3'; ATF3, 5'-CCT ATGCAAAGCAGGATCCC-3', 5'-GCGTTGTCAGCCACAGTGG-3'; GRP94, 5'-CTGGGTCAAGCAGAAAGGAG-3' 5'-TCTCTGTTGCTTCCCGACTT-3'; BiP, 5'-GCTTCGTGTCTCCTCCTGAC-3', 5'-TAGGAGTCCAGCAACAGGCT-3'; Chop, 5'-CCAACAGAGGTCACACGCAC-3', 5'-TGACTGGAATCTGGAGAGCGA-3'; Xbp1-spliced, 5'-GAGTCCGCAGCAGGTG-3', 5'-GTGTCAGAGTCCATGGGA-3'; Xbp1-total, 5'-CACCTTCTTGCCTGCTGGAC-3', 5'-GGGAGCCCTCATATCCACAGT-3'*. The rat primer sequences were as follows: *Actin, 5'*-ATC CTG GCC TCA CTG TCC AC-*3', 5'*-CTA GAA GCA TTT GCG GTG CA-3'; *GAPDH, 5'*-CACCACCAACTGCTTAGCCC-*3', 5'*-TGGCATGGACTGTGGTCATG-3'*; Insulin I, 5'-CAGCACCTTTGTGGTCCTCA-3', 5'-CCCACACACCAGGTACAGAGC-3'; Insulin II, 5'-CTGCCCAGGCTTTTGTCAAA-3', 5'-CTTCCACCAAGTGAGAACCACA-3'; MafA, 5'-GGCACATTCTGGAGAGCGA-3', 5'-CCCGCCAACTTCTCGTATTTC-3'; Pdx-1, 5'-CCACCAAAGCTCACGCGT-3', 5'-CTGCGTATGCACCTCCTGC-3'; NeuroD, 5'-GCTTGAAGCCATGAATGCAG-3', 5'-TCCTCTCCCCCATTTCTCAGA-3'; ERp72, 5'-TCTAACCAATCACCGGGCTG-3',5'-TCATGGTAAGGTGCCGAGG-3'; BiP, 5'-ACCCTTACTCGGGCCAAATT-3', 5'-AGAGCGGAACAGGTCCATGT-3'; ERp58, 5'-CGAAAACTTCGAGAGTCGCG-3', 5'-GCAAGCCTCTTGCAATGTCC-3'; ERp57,, 5'-GGACTCAAG CGAAGTGACGG-3'; 5'-TCTGCTGCCAGCAAGAACTG-3'; Ero1 L, 5'-TTCACTGAGGAGGGCGGTT-3', 5'-CAGGACGGTCACTGCAATCA-3'; Ero1 beta, 5'-GTGCTTTGTCAAAGGTGG CC3'; 5'-GCAGAAGGGTCTTGGTGTCAG-3'; Crebpp1, 5'-ACGCCGGAGTCAATTCCTATC-3', 5'-GAGCTGATGTTGCGG GAAGA-3'; Psma5, 5'-TCACACCCCTGTCGTACTCG-3', 5'-TTTCAGTCGTGTGGCCTTTG-3'; Sec61a, 5'-GCTTCTGAATTT CCGGCAAG-3', 5'-AGGCAGGGAGTGTAGTCGGAC-3'; Sec63, 5'-TGGGTGAGTGAGACCTTCCC-3', 5'-AACCCCGGATCTTCCCAGTA-3'; Der1, 5'-CACCAGCCATGCTAAGCAGA-3', 5'-TCAGTGTGGGTCAGGTCCAAG-3'; Herpud1, 5'-GTGCTCTGTTGCTGGAGGCT-3', 5'-AGCACATCGTCATCCTGTGG-3'; Xbp-1 spliced, 5'-CTGAGTCCGAATCAGGTGCAG-3', 5'-ATCCATGGGAAGATGTTCTGG-3'; Xbp-1 total, 5'-CCCTTCTCCCTTCAGCGAC-3', 5'-CGTTGGCAAAAGTGTCCTCC-3'; Rat cyclin D2, 5'-TGCTGACCAAGATCACCCAC-3', 5'-CCTGGCAGGCTTTGAGACAA-3'; Rat cyclin B2, 5'-GCCCCTGAGGATGTCTCCAT-3', 5'-AGAGAAAGCTTGGCAGAGGCT-3'; Rat cyclin A2, 5'-AGTGTGAAGATGCCCTGGCT-3', 5'-TGGCTCCGGGTAAAGAGACA-3'; Rat cyclin E, 5'-GTCGCAGGGTTGCTGTTGAT-3', 5'-CATGCTTGCTCACGACCACT-3'*

### Western blots

Total 832/13 cellular protein was extracted with RIPA buffer (1% Nonidet P40, 0.5% sodium doxycholate, 0.1% SDS, 1 × PBS, pH 8.0) containing 1× protease and phosphatase inhibitor cocktails (Sigma, Inc.) Protein expression was assayed by Western blots. Primary antibodies used in the analysis were: ERp72 (1:2500, Stressgen, Inc), GRP78/BiP (1:500, Santa Cruz, Inc), ERp57 (1:300, Santa Cruz), and anti-GFP (Sigma, Inc.) to detect EFYP-ATF6. For Western blots performed on islets from single mice, 60 islets from each mouse were dissolved in 2× SDS sample buffer and then loaded onto a 4-15% gradient gel.

### Protein synthesis

After 16 or 24 hr post-transduction, 832/13 cells were deprived of methionine and cysteine (Met/Cys) for 30 minutes at 37°C in Met/Cys-free DMEM, 10% dialyzed FBS. The cells were then labeled with [35S] Met/Cys (500 μCi/ml) at 37°C for 0, 15, 30 minutes, and the reactions were stopped by the addition of concentrated non-radioactive Met/Cys solution (0.1 M each). After two washes with PBS, the cells were lysed in RIPA buffer and then total cellular protein was precipitated with 10% trichloroacetic acid (TCA). The precipitates were washed with 20% ice-cold acetone, air-dried for 20 minutes and then dissolved in 30 mM Tris, 7 M urea, 2 M thiourea, and 4% CHAPS. The resident radioactivity was measured by scintillation counting and normalized to total protein content.

### Immunohistochemistry

Paraffin-embedded sections or cryo-sections were subjected to immunohistochemistry using the following primary antibodies: ERp72 (1:1000, Stressgen), insulin (1:500, Linco Research); proinsulin (1:1000, Ole D. Madsen, Beta Cell Biology Consortium); GRP78/BiP (1:500, Santa Cruz). Appropriate secondary antibodies conjugated with Alexa Fluor350, 488 or 555 dye (Molecular Probes) were used to visualize the labeled cells. Fluorescence images were captured and analyzed with a Nikon Eclipse E1000 and Image-Pro Plus (Phase 3 Imaging Systems, GE Healthcare, Inc.).

### Determination of the oxidative state of ERp72 and ERp57

At 24 hours post-transfection, the medium was removed, and the cells were briefly washed with ice cold PBS and lysed at 0°C for 5 min in 20% formic acid/2% SDS. Cell lysate proteins were precipitated with 10% TCA. Precipitates were washed twice with ice cold 70% acetone. The TCA precipitates were resuspended in 80 mM Tris-HCl pH 6.8, 2% SDS, supplemented with a cocktail of protease inhibitors with or without 10 mM 4-acetamido-4'-maleimidylstilbene-2,2'-disulfonic acid (AMS, Molecular Probes, Inc.). After incubating for 30 min at room temperature and then for 10 min at 37°C, samples were subjected to non-reducing SDS-PAGE followed by western blot analysis to detect ERp72 and ERp57. Non-transfected cells were treated with 10 mM DTT or 5 mM diamide for 10 min at 37°C as reduced and oxidized controls.

## List of Abbreviations

(ERAD): endoplasmic reticulum associated protein degradation; (eIF2α): eukaryotic translation initiation factor-2 alpha.

## Authors' contributions

DF carried out the cellular and molecular biology experiments and wrote the original draft of the manuscript. JW performed the protein synthesis experiment. BM provided management of the laboratory in which all experiments were conducted and edited the manuscript. SG performed the ATF6 experiments. DC and DF designed the experiments, and DC analyzed and interpreted the data and edited the manuscript. All authors read and approved of the final manuscript.
